# Understanding the Work-Related Roles in the Work–Personal Life Reconciliation of Nurses in Intensive Care Units: Constructivist Grounded Theory Research

**DOI:** 10.3390/healthcare13172134

**Published:** 2025-08-27

**Authors:** Miguel Valencia-Contrera, Lissette Avilés, Naldy Febré

**Affiliations:** 1 Faculty of Nursing, Andrés Bello University, Santiago 8370146, Chile; naldy.febre@unab.cl; 2 Nursing Studies Department, University of Edinburgh, Edinburgh EH8 9AG, Scotland, UK; lissette.aviles@ed.ac.uk

**Keywords:** work–life balance, nursing, Intensive Care Units, qualitative research, grounded theory

## Abstract

**Objectives**: To theorize the work-related dimension involved in the process of reconciling work and personal roles among nurses working in Intensive Care Units. **Methods**: Constructivist grounded theory was employed to conceptualize the phenomenon of interest from the perspectives of nurses, their families, and administrative staff. Data were derived from 202 h of non-participant observation in two high-complexity hospitals in Chile (one public and one private), 57 institutional documents, and 51 in-depth interviews. Data analysis followed the constant comparative method and multilevel coding. To ensure methodological rigor, the study adhered to the 13 criteria for constructivist grounded theory research proposed by Charmaz and Thornberg and was approved by a scientific ethics committee. **Results**: Work-related roles are defined as the set of behaviors associated with the professional context, which are shaped by nine sources of interaction: (1) Job content; (2) Workload and pace; (3) Work schedule; (4) Control; (5) Environment and equipment; (6) Organizational culture; (7) Interpersonal relationships; (8) Function within the organization; and (9) Career development. **Conclusions**: The study presents the theorization process of the work-related dimension involved in the reconciliation of work and personal roles among nurses in Intensive Care Units. Work-related roles are essential to understanding this reconciliation process. The findings provide evidence for the existence of nine sources of interaction, which are influenced by institutional strategic planning and, in turn, by national and international public policies.

## 1. Introduction

Work–family interaction is defined as “*a process in which a worker’s functioning (behavior) in one domain (e.g., home) is influenced by (negative or positive) load reactions that have built up in the other domain (e.g., work)*” [1]. It is recognized as an occupational psychosocial risk with particular characteristics, such as being the only one that considers the worker’s personal and family aspects, yet it remains among the least explored [2].

The importance of studying this phenomenon lies in the numerous and diverse negative associations that emerge when the work and personal domains come into conflict, affecting not only workers and their families but also organizations and society at large [3]. On the organizational level, negative outcomes include impaired job performance, absenteeism, and turnover intention. At the family level, consequences include strained relationships, parental conflict, and irritability in parenting. On a personal level, depressive symptoms, stress, and deteriorated physical health have been documented [4,5,6,7].

The International Labour Organization has emphasized the importance of adequately managing these occupational risks to mitigate their harmful effects, which were exacerbated during the COVID-19 pandemic [8]. In Chile, the government monitors these risks using the Copenhagen Psychosocial Questionnaire (COPSOQ), with the Superintendence of Social Security (by its Spanish acronym, SUSESO) being responsible for publishing the results. According to the latest 2024 report, in the “work–life balance” dimension, the economic sector with the highest percentage of suboptimal risk (medium and high risk) was the healthcare sector, with 89.7% [9].

In the healthcare field, nursing has been identified as a particularly vulnerable group [10,11]. This vulnerability can be partly explained by hostile working conditions that hinder the ability to provide family care [12], as well as by the predominantly female composition of the profession [13], which constitutes approximately 90% of the global nursing workforce according to the WHO [14]. Given the wide range of nursing roles, it is expected that certain practice areas are more exposed to this occupational risk, with Intensive Care Units (ICUs) being particularly conflictive compared to general wards [15,16,17].

Despite the significance of the phenomenon, knowledge gaps persist, limiting its understanding and appropriate intervention. One such gap is the lack of clarity regarding the work-related elements involved in the work–personal life interaction. A recent literature review underscored the need for future research to explore the organizational factors associated with this phenomenon [6].

### 1.1. Organizational Factors Involved in the Work–Personal Life Interaction

The earliest conceptualizations of the sources of work–life interaction can be traced back to Cooper and Marshall’s job stress model [18], which identified five sources of stress: intrinsic to the job, role in the organization, career development, workplace relationships, and organizational structure and climate. Later, Kasl proposed ten sources, some of which remained while others were revised or merged [19,20]. These ideas strongly influenced Cox’s classification [21,22,23], which grouped them into the following: organizational function and culture; organizational role; career development; decision-making autonomy; interpersonal relationships at work; home–work interface; work environment and teams; task design; workload and pace; and working hours.

Cox’s final model has become one of the most influential in the field [24], widely recognized by the scientific community and endorsed by international organizations [25,26,27,28,29,30]. The sources of workplace interaction are in constant interplay and may exert a synergistic negative effect. For instance, a conflict in one domain can compromise others, thereby amplifying its impact on the worker. Conversely, when some sources are perceived as positive, they may buffer the negative effects of others [31].

These interactions have been primarily described by two influential models: the Demand-Control-Support model and the Effort–Reward Imbalance model. Initially, Karasek proposed the “Job Strain Model” [32], positing that high demands coupled with low control act as stressors. Later, Johnson highlighted the importance of social support [33], which Karasek had not originally included. This led to the expanded model by Johnson and Hall [34], incorporating social support as a moderating variable. According to the model, job strain results from the interaction of psychological demands (e.g., workload, time pressure, and role conflict), control (e.g., decision-making autonomy and skill use), and social support.

On the other hand, Siegrist developed the “Effort–Reward Imbalance Model” [35], grounded in sociology and social psychology, and influenced by human stress theory [36]. This model posits that the efforts invested (quantitative, qualitative, and physical demands of the job) must be adequately matched by the rewards received (e.g., recognition, esteem, salary, promotion opportunities, and job security).

### 1.2. Theory of Work–Personal Role Reconciliation

Given the numerous and varied theoretical gaps in the study of work–family interaction, which limit the understanding and effective management of the phenomenon—particularly among vulnerable groups such as critical care nursing professionals—we recently developed the Theory of Work–Personal Role Reconciliation [37]. This theory explains the process of reconciling work and personal roles among ICU nurses from the perspectives of the nurses themselves, their families, and their nursing supervisors.

Role reconciliation is described as the basic social process that explains the interaction between nurses’ professional and personal roles. This process unfolds through three stages: resisting the war of roles, hitting rock bottom, and reconciling.

In the original publication, we described the development of the core of the theory—the basic social process. However, due to the breadth and complexity of the phenomenon, it was not possible to provide a detailed account of the configuration of work roles, including their sources of interaction, characteristics, and relationships. Therefore, the present study aims to theorize the work-related dimension involved in the process of reconciling work and personal roles among nurses working in Intensive Care Units.

## 2. Materials and Methods

### 2.1. Study Design

Given the complex nature of the phenomenon—and based on the premise that all complex social events are composed of social processes [38]—a grounded theory design was selected, as it allows for an in-depth understanding of social processes and enables the capture of their depth and theoretical potential [39].

Sociologist Kathy Charmaz states that this type of design enables the conceptualization and often the unveiling of invisible social processes [40]. Constructivist grounded theory (CGT) understands analysis as interpretative rather than as an objective report or a singular view of the studied phenomenon. Consequently, the researcher is more aware of the relativity of the empirical world, considering multiple realities and perspectives [41].

In this regard, constructivist grounded theorists assume that the values, priorities, positions, and actions of the observer influence their interpretation of the data [42]. CGT is inductive and adopts strategies from classic grounded theory [43]; however, it is distinguished by four key elements [40]: adoption of a relativist epistemology; acknowledgment that both the researcher and the participants hold multiple perspectives, roles, and realities; a reflective stance toward the researcher’s own background, values, actions, research contexts, relationships with participants, and representations of them; and grounding the research within the historical, social, and situational conditions under which it was produced.

In addition, symbolic interactionism was employed as the methodological and theoretical framework [44], providing a worldview and language suitable for conducting CGT studies [45]. This study was reported following the Consolidated Criteria for Reporting Qualitative Research (COREQ) checklist [46] [Supplementary Materials File S1]. For further details regarding the methodological decisions of the study, readers are referred to the research protocol [47].

### 2.2. Researcher Positioning

Given the adoption of a constructivist stance in the development of this study, it is essential to acknowledge the position of the researcher, as the theorization emerged through co-construction between the researcher and the participants. The principal investigator (MVC) is a male nurse and academic affiliated with a university in Chile. He has clinical experience in critical care units, including during the COVID-19 pandemic. His initial exposure to occupational health and safety began during his undergraduate nursing training, which later led him—during his postgraduate studies—to deepen his focus on psychosocial risks at work. Subsequently, during his doctoral studies, he concentrated on the work–family conflict. Therefore, his clinical and research experiences in this field provided him with theoretical sensitivity in the co-construction of the proposed theory.

### 2.3. Recruitment

The recruitment of participants was carried out in Chile, a country located on the southwestern edge of South America, with an estimated population of 18 million inhabitants distributed across 16 regions [48]. Chile has a mixed healthcare system, comprising both public and private institutions within its care network [49].

For this study, two institutions were considered—one public and one private—anonymized as H1 and H2. Recruitment began at H1 using purposive sampling and continued at H2 following an iterative process guided by theoretical sampling.

Prior to data collection, the principal investigator presented the study at both H1 and H2, where initial contact with participants was established. The researcher introduced himself and addressed any questions regarding the research.

Inclusion criteria encompassed ICU nurses, nurse administrators, and adult family members of nursing professionals. Nurses who were on medical leave or absent from their workplace for any reason at the time of data collection were excluded from the study.

### 2.4. Data Collection

From the theoretical–methodological orientation of symbolic interactionism adopted in this study, it is assumed that society, reality, and the self are constructed through interaction [44], and therefore are dependent on language and communication [50]. Data were collected through non-participant observation, in-depth interviews, and document analysis—methods recommended for the development of grounded theory studies [51,52]. Data collection was carried out by the principal investigator (MVC). Data collection began upon receiving approval from the scientific ethics committees and continued throughout the year 2024.

Data collection began at H1 with non-participant observations, guided by the following central question: how does the work–family interaction process unfold among ICU nursing professionals? Field notes were taken in the researcher’s notebook, later digitized and analyzed. The information gathered enabled the identification of the physical and social characteristics of the environment, individual and collective actions, as well as the consequences of social behaviors [53].

Moreover, the observations informed the in-depth interviews. These interviews were conducted with “experts” on the phenomenon—namely, individuals who had either experienced the phenomenon directly or had observed others who had experienced it [54]. In this context, ICU nurses, nurse coordinators, and adult family members of nurses were included. The individual interviews lasted an average of 53 min and were audio-recorded and transcribed verbatim for subsequent analysis. In most cases, interviews were conducted face-to-face at H1 or H2, in a private room arranged for this purpose. If privacy could not be guaranteed, an alternative location was coordinated with the participant.

No interviews required repetition, and transcripts were not returned to participants, primarily because data analysis was conducted iteratively, following grounded theory procedures, which allowed for the refinement, verification, and cross-checking of information during the course of data collection, employing data triangulation rather than direct validation with participants. The data collection format is available in [Supplementary Materials File S2], which was structured based on established recommendations [50,55,56].

Finally, documents relevant to the field were considered as data. These documents facilitated the identification of the situational, cultural, social, economic, historical, and political contexts [50], and were thus used to analyze the organizational and political levels in which the phenomenon is situated. Examples of documents analyzed include the following: international ILO conventions [57,58], the Chilean Constitution [59], labor laws [60,61], health regulations [62,63], ministerial guidelines for psychosocial risk management at work [64], ministerial guidelines for ICU organization and operation [65], and design guidelines for ICU hospital facilities [66].

### 2.5. Data Analysis

The data analysis process began alongside the initial stages of data collection, employing an initial coding phase. This was followed by focused coding and categorization, and ultimately culminated in theoretical construction. The analysis primarily consisted of multilevel coding and constant comparative methods [45]. Additionally, memo writing was used throughout the process, serving as a tool for recording reflections, interpretations, analytical questions, and instructions for further data collection [67]. This process required continuous interrogation of the data in light of the emerging analysis.

The coding procedures varied depending on the data source: incident-by-incident coding for observational data, line-by-line coding for interview transcripts, and word-by-word coding for documentary materials [68]. After initial coding, those codes with greater analytical power were identified, or new codes were developed, with the aim of increasing the level of abstraction. Explanatory reasoning—abduction—was employed for theory development, allowing for the progression from concepts to a theoretical formulation [69]. Data management and analysis were conducted using ATLAS.ti version 25 (ATLAS.ti GmbH, Berlin, Germany).

The category “work roles” emerged as the construct explaining the influence of the work domain in the interaction between workers’ professional and personal spheres. Pattern repetition was considered the saturation point for this study [50]. The coding process used to develop the “work roles” category is presented in Supplementary Materials File S3.

**Figure 1:**
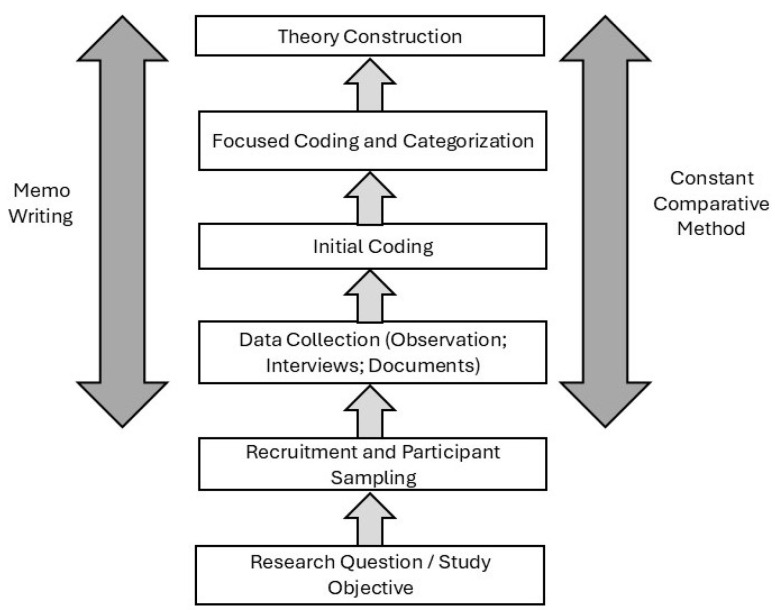
Summary diagram of the research process.

Figure 1 below presents a diagram summarizing the overall research process of the study, while preserving the essence of Charmaz’s original proposal [45].

### 2.6. Rigor and Trustworthiness

The rigor and trustworthiness of the study were ensured through the analysis of participants’ lived experiences, which guided the theoretical construction. Additionally, the use of gerunds and in vivo codes helped to focus attention on the process and reveal meanings that accurately reflected the reality and experiences of nurses, nurse administrators, and family members. The criterion used to conclude the grounded theory was theoretical transferability [54], which ensures that the emerging concepts and theory possess applicability beyond the specific context and situation in which they were initially identified. This was supported by the recruitment strategy involving two institutions—one public and one private—within a local context characterized by a mixed healthcare system. Thus, the level of abstraction achieved allowed the findings to transcend the particularities of H1 and be transferred to the context of H2.

Furthermore, the development of this study adhered to the 13 rigor criteria for constructivist grounded theory (CGT) proposed by Charmaz & Thornberg [70]. The fulfillment of each criterion is detailed below: 1. Methodological self-awareness was maintained through continuous memo writing and the justification of methodological decisions. 2. The study was supervised by experienced researchers (LA and NF), and foundational literature in the field significantly influenced methodological decisions. 3. A critical literature review was conducted and referenced throughout the manuscript. 4. Valuable data were collected, focusing on the main actors involved in the interaction of ICU nurses’ work and personal roles. 5. All decisions were clearly justified and made transparent. 6. The process of data collection and analysis was iterative. 7. Ambiguities were acknowledged in memos and addressed during supervision sessions. 8. Throughout the research process, analytical questions that prompted abductive reasoning were developed; an example is provided in Supplementary Materials File S3. 9. Theoretical explanations of the data were sought and articulated in the Results and Discussion sections. 10. Sufficient data were gathered to enable comparison and the creation of analytical categories. 11. Constant interrogation of the categories enabled justification of their properties and theoretical scope. 12. The emerging theory was considered provisional, allowing for changes and adjustments as the analysis progressed. 13. Finally, the findings were discussed in relation to the current state of the art in the field.

### 2.7. Ethical Considerations

The study was reviewed and approved by three ethical–scientific committees: one associated with the sponsoring institution and the other two affiliated with the participating institutions, as required by their respective regulations. The initial review was conducted by the Ethics Committee of the Faculty of Nursing at Universidad Andrés Bello on 3 April 2024, under the code “L4/CECENF/01-2024,” followed by approval from the ethics committee of the public institution under code “10/2024,” and the private institution under code “162-11-24.”

Prior to each interview and observation, informed consent was obtained both orally and in writing. Confidentiality was ensured by assigning a code to each participant and omitting any details that could potentially reveal their identity.

## 3. Results and Discussion

The results and discussion of this study will be presented jointly, with the aim of facilitating the integration of data, state-of-the-art knowledge, and the theoretical literature consulted. This structure also allows for an explicit exposition of the abductive reasoning process [71]. The data analyzed in this study comprised 202 h of observation, 57 documents, and 51 in-depth interviews. Table 1 presents the demographic data of ICU nurses and nurse administrators, while Table 2 presents the demographic data of the nurses’ family members.

**Figure 2:**
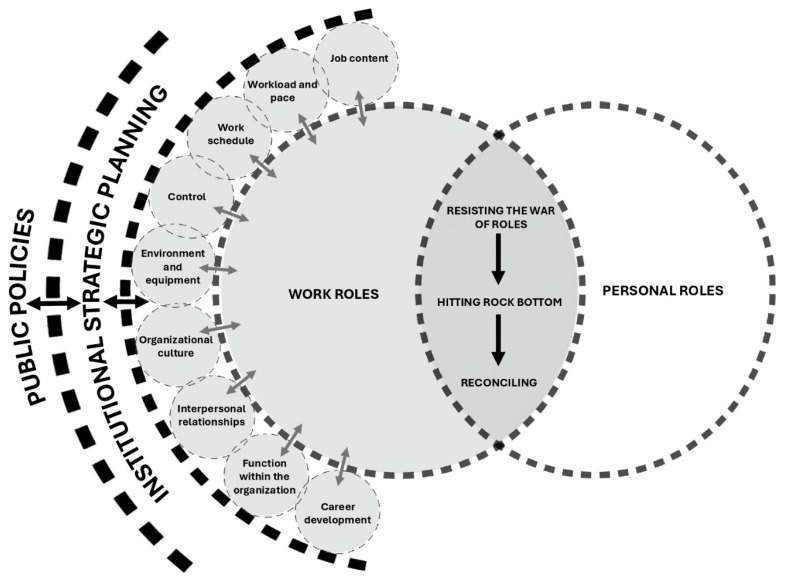
Process of reconciling work and personal roles.

“Work roles” are part of the occupational sphere of ICU nurses and serve to theoretically explain their experiences as they come into contact with various sources of work-related interaction. These roles are essential for understanding the process of “reconciling work and personal roles” as the basic social process (BSP) (see Figure 2).

The report on the basic social process (BSP) and personal roles has been published separately due to the complexity of the phenomenon [37]. Therefore, the present report focuses specifically on the theorization of work roles. Work roles are defined as the set of behaviors associated with the occupational context of ICU nurses—an adaptation of Biddle’s definition of roles [72]. Nurses’ behaviors within the work sphere are shaped by multiple sources of interaction, which are themselves influenced by institutional strategic planning, and, in turn, by both local and international public policies [73].

Historical contributions in the field were instrumental in understanding the phenomenon in all its complexity and were crucial for theorizing and explaining the work-related sources of interaction that influence the reconciliation of work and personal roles among ICU nurses [18,21,22,23,24,32,33,34,35,36]. Each source of interaction is described in detail below.

### 3.1. Job Content

Job content encompasses job tasks, skill utilization, task variety, emotional demands, and cognitive demands [30].

*“I feel that this job… makes you reflect on many things, it makes you question many things, you know? Many, many, many… because, ultimately, you’re working with life and death every day—with sorrow, with pain, with need…”.*

(Interview, Nurse Administrator 1, ICUp, H1)

In the specific context of ICUs, care is centered on critically ill patients who require a team with high levels of knowledge, intense concentration, emotional control, and both psychomotor and cognitive skills. These professionals must perform complex tasks and respond to health problems requiring immediate action [74,75,76].

*“You learn to prioritize in these units because you’re always walking on the edge, at the edge of an abyss—between life and death…”.*

(Interview, Nurse Administrator 2, ICUa, H2)

*“We take care of everything—respiratory, nutrition… we cover it all. Even things like making sure the unit doesn’t run out of soap…”*.

(Interview, Female Nurse 16, ICUa, H1)

The state of the art in this field acknowledges that nursing in ICU settings involves a highly demanding and specialized role [77]. Due to the nature of the work, nurses are under constant exposure to emotional stress and to the process of patient death [78]. For example, reviews in this area reveal the complexity of care actions performed by nurses after a patient’s death and the profound personal impact of these experiences [79,80].

Moreover, participants highlighted that the demanding nature of ICU work often requires ongoing study and professional development, which negatively affects their personal time and ability to meet the challenges of their role.

*“The ICU demands extra time even when you’re at home—because you have to study, stay updated, prepare yourself… I need to study at least two hours so I can then spend time with my family, find some balance. So yes, I do feel that being here is more demanding…”.*

(Interview, Female Nurse 28, ICUa, H2)

### 3.2. Workload and Pace

Workload can be both quantitative and qualitative in nature: quantitative workload refers to the amount of work, while qualitative workload pertains to its complexity. Work pace, in turn, refers to the demands related to the speed and deadlines with which tasks must be completed [23]. Additionally, in the current context, technological demands in the workplace significantly contribute to workload [30].

*“It’s a lot of work, but I’ve more or less gotten used to it—especially the routine. At first it was difficult, especially with the IT system. I came from working in public and private institutions where everything was done on paper. So the electronic system was really challenging at first—it took me a couple of months, I’d say…”.*

(Interview, Male Nurse 25, ICUa, H2)

Reviews in the field have indicated that nurse-to-patient ratios are often used as the primary indicator to assess ICU workload. However, the construct is more complex than simply the number of nurses per patient [81], as workload involves both quantitative and qualitative aspects. In ICU settings, nurses are continuously exposed to high levels of workload [78,82,83], from highly visible tasks associated with medical procedures to information-based work that requires reviewing, processing, and acting on numerous data points while caring for multiple patients simultaneously [84]. Notably, workload tends to increase during the first days of hospitalization, during the dying process, and at the start of shifts [85,86].

*“In nursing, we also carry more workload… I don’t know how to explain it, but it’s like a psychological workload. We have to be more aware of many things compared to other professionals. So, because I need to stay on top of what’s happening with one patient, and then the other, I end up alerting the dietitian or the physician… they help, but I’m the one who has to stay aware. I think that really does affect how we’re able to balance roles with the family and everything”.*

(Interview, Female Nurse 21, ICUa, H2)

### 3.3. Work Schedule

Work schedule refers to the duration, flexibility, and type of work shifts [87], as well as the presence of multiple job holdings [30]. Two characteristics of scheduling are particularly conflictive: shift work and extended work hours [23]—both of which are prevalent in the nursing profession [14].

*“The shifts are very long. And on top of that, we often take extra shifts, so the commute adds another three hours to the job. That makes it 15 hours in a 24-hour day… it’s hard…”.*

(Interview, Female Nurse 8, ICUa, H1)

Preferences regarding shift duration are a matter of ongoing debate. For instance, the study by Ose et al. [88] described the diverse preferences of nurses regarding 12-h shifts, which were influenced by personal health, family responsibilities, tolerance to workload, sleep problems, and personality traits—highlighting the importance of voluntary choice in shift assignment. In contrast, the study by Webster et al. [89] analyzed the impact of shift length and concluded that most respondents believed 12-h shifts would benefit their health—a belief supported by official records indicating a reduction of 69 sick leave days and 216 family leave days.

*“I think working 12-hour shifts, working at night, is not something that facilitates family life—because sometimes you can’t be there for Christmas, New Year’s, your child’s birthday, your husband’s birthday, or family celebrations. And you also can’t be there during difficult moments, like when your child is sick, or your mother is sick, you know? So, it’s not a job that helps with family life, right? I think you have to be very patient to be the partner of someone who works 12-hour or sometimes even 24-hour shifts… your children miss you, your husband needs you, and the house needs you too—because, generally, it’s us women who manage the household, isn’t that right?”.*.

(Interview, Nurse Administrator 2, ICUa, H2)

*“I never leave at 8 o’clock sharp. I always leave at 8:30”.*

(Interview, Female Nurse 29, ICUa, H2)

### 3.4. Control

Control refers to the extent to which workers can participate in decisions that affect their work [21], such as control over workload, tasks, methods, work location, and pace [30].

*“Sometimes you ask yourself, what am I walking into tomorrow? Like, what will be there? And you start to… I don’t know, even get anxious just from not knowing. During the pandemic it was the same—like, what am I walking into? What kind of disaster might be waiting?”.*

(Interview, Male Nurse 20, ICUa, H2)

In ICU settings, uncontrollable workloads have been documented [90]. A qualitative study highlighted several contributing factors, including the presence of multiple physicians during work shifts, which required nurses to interact with each physician individually in order to receive a response. This delayed access to critical information. In addition, newly arrived medical residents often lacked the necessary knowledge or experience, which negatively affected information transfer. Illegible handwriting was also cited as a barrier [91]. All of these factors contributed to a lack of control over work conditions.

*“If I fall behind, I get stressed—I like to do things properly. And if I don’t do them properly, I feel bad… When I used to work during the day [a Monday-to-Friday shift from 8:00 to 17:00], if I fell behind, I could catch up the next day… It’s a different kind of stress—more manageable, I think”.*

(Interview, Female Nurse 3, ICUa, H1)

*“During the pandemic I had to cover another ICU… I think this unit is the most complicated of all… In the other unit, for example, we would do rounds and they actually considered our input…”.*

(Interview, Female Nurse 38, ICUp, H2)

### 3.5. Environment and Equipment

The environment and equipment dimension includes physical working conditions, environmental factors, safety conditions, work equipment, use of technology, and work modalities [30].

*“I remembered when I used to be one of them—it was strange. I felt reflected in them. I felt a rush of adrenaline, a bit overwhelmed, with tachycardia; I think it was the combination of monitors, ventilators, alarms, and people performing procedures…”.*

(Field Notes, ICUa, H1)

**Figure 3:**
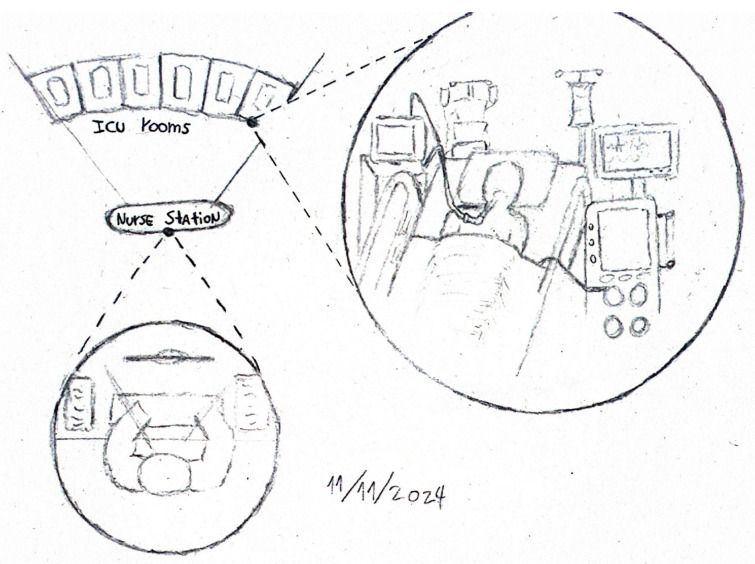
Field notes from observations.

ICUs are equipped to care for critically ill patients who require continuous monitoring of vital signs and complex therapies, making the use of medical technology unavoidable. Observations conducted in this study (see Figure 3) identified several elements that shaped the context under analysis. These included the sounds of mechanical ventilators, monitor alarms, continuous infusion pumps, and renal replacement therapy machines. Other notable environmental factors included lighting, ventilation, temperature, and even odors, which emerged as significant sources of interaction. Finally, the ergonomic characteristics of the physical environment, such as the location and setup of clinical workstations, also played a role—as illustrated in Figure 3.

Recent studies have concluded that machines in ICU settings can contribute to reducing mental workload [84]. However, other reports highlight that human–machine interaction may also have detrimental effects on healthcare workers—for example, alarm fatigue [92,93,94], which negatively impacts nurses’ well-being and, consequently, their personal roles.

*“I think it really depends on the person, because, for example, when I go home to rest, I sometimes still hear the sound of the ventilator [touches her ears]… it really depends on the person. Personally, I try to forget about work a little, but sometimes it just comes back…”.*

(Interview, Female Nurse 1, ICUa, H1)

### 3.6. Organizational Culture

Adapting the definition proposed by Aldhafeeri [95] and Leka & Jain [30], organizational culture is understood as a unique work environment shaped by values, beliefs, a climate of psychosocial safety, and leadership, which guide management practices, communication processes, behaviors, and attitudes within the organization. The data indicated that an organizational climate that promoted psychosocial well-being was not always present.

*“There is a high demand for extra shifts here, because they impose them on you, they force you, since there is not enough staff. So, those of us who are here have to cover. These are like job positions that do not officially exist, because the Ministry hasn’t authorized them, you understand?”.*

(Interview, Female nurse 7, ICUa, H1)

Several practices were identified that hindered the reconciliation between nurses’ professional and personal roles. These included the imposition of additional shifts, barriers to requesting shift changes, limited support across professional levels, and a lack of strategies—even when feasible—to address the constant exposure to highly emotionally demanding situations.

*“In one specific case, a relative of mine died from COVID-19, and when I informed my supervisor, the reaction was something like: ‘Well, nothing we can do!’ No support at all—despite the fact that we work with COVID-19 patients every single day. So, those things—those little things—leave a mark. You start thinking, Why should I give so much when it’s not reciprocated?”.*

(Interview, Male nurse 15, ICUa, H1)

*“At the very least, I tell them [referring to charge nurses], if there’s a very critical patient and, I don’t know, someone recently experienced a loss, try not to assign them to that patient. Switch them with someone else if you can, because you know those are wounds that need time to heal, and facing the same thing repeatedly just reopens them…”.*

(Interview, Nurse Administrator 8, ICUa, H1)

Four types of organizational cultures have been described: bureaucratic, clan, entrepreneurial, and market cultures. From this conceptual framework, organizations may exhibit different combinations of these cultural types. On one hand, the bureaucratic culture emphasizes formality, procedures, rules, standardization, and hierarchical structures. Secondly, the clan culture values tradition, commitment, loyalty, socialization, and teamwork, and therefore requires transcending the simple exchange of labor for salary. Thirdly, the entrepreneurial culture is characterized by creativity, dynamism, risk acceptance, and a commitment to innovation, experimentation, and change promotion. Finally, the market culture aims at achieving measurable and desirable goals [96].

The state of the art in ICU settings highlights the critical role of leadership [97], which is regarded as essential for establishing and maintaining a healthy work environment [98]. It has been observed that units with strong cohesion and a participatory environment tend to achieve greater work engagement among nurses [99].

### 3.7. Interpersonal Relationships

Interpersonal relationships encompass the quality of work relationships with managers, colleagues, clients/service users, teamwork, social support from managers and colleagues, and policies and procedures for managing conflict, diversity, and inclusion [30].

*“So, it’s like a very lonely position [referring to charge nurses]. I feel like there’s not much support, and those kinds of things do have an impact…”.*

(Interview, Nurse Administrator 3, ICUa, H1)

Traditionally, this source of interaction has described supervisors, subordinates, and colleagues as the primary actors [18,21]. More recently, the role of clients/service users has also been highlighted [30]. However, the present study emphasizes the need to explicitly consider patients’ family members within healthcare services, as they play a prominent role as sources of interaction.

*“…and one day the patient passed away. It was very sad. The family invited me to the wake, the funeral—it was far away, I don’t remember where—but I felt deeply affected, because there was a relationship with them [family members of the deceased patient], and it made me very sad, very sad. There were days when I had to face them, say goodbye… and saying goodbye meant something to me too”.*

(Interview, Female Nurse 37, ICUp, H2)

In the ICU context, interpersonal relationships have been described as enriching for nurses, both through interactions with coworkers and feedback from patients and their families [100]. However, this source of interaction can also have negative effects, ranging from being overwhelmed by repeated questioning from patients’ relatives—often due to inadequate communication from other team members [91]—to exposure to physical aggression [75]. On the other hand, supervisor support is fundamental [101]. In some cases, a laissez-faire leadership style has been described, characterized by little effort to meet nurses’ needs, lack of feedback, delayed decision-making, and minimal staff involvement [102].

*“Always, always, always I’ve been able to talk about things with them [my coworkers]. There’s never been a time when I’ve had to keep sadness or anger bottled up. I generally try to talk things over with them because they’re the ones who support me every day. So, if something has happened or I’ve had a tough situation, the team has been amazing [‘a seven’—a local expression meaning excellent]. Since we all have such a good relationship, I feel like we’ve always supported one another…”.*

(Interview, Female Nurse 34, ICUp, H1)

### 3.8. Function Within the Organization

Function within the organization involves clarity in the definition of roles and responsibilities at work [87], as well as the establishment of goals and professional identity [30]. Four key conditions are relevant when analyzing this source of interaction: role ambiguity, role conflict, role insufficiency, and responsibility [23]. Ambiguity occurs when the worker lacks adequate information regarding their role. Conflict arises when the individual is required to perform duties that contradict their values or when multiple responsibilities are incompatible with one another. Insufficiency refers to the organization’s inability to fully utilize the worker’s skills and capacities. Responsibility may involve accountability for others and/or for things such as equipment, budgets, or other resources [18].

*“It’s very dynamic. We have certain established routines… but we adapt according to the service’s needs”.*

(Interview, Female nurse 6, ICUa, H1)

*“The previous coordinator left, and I was left in charge… so it was like, overnight, I became the boss. It was a very sudden change, and at the beginning I tried, obviously, to respond well—to respond well and do things just as well or better than the previous supervisors. That caused me a lot of stress at first”.*

(Interview, Nurse Administrator 7, ICUp, H1)

The data revealed that role clarity was not present in all cases, particularly among newly hired workers with limited orientation processes and those transitioning from clinical to leadership positions. This aligns with the theoretical perspective of Martha Alles, who argues that the time invested in the induction of a new employee is a fundamental component of the future working relationship. At a minimum, this should include information about the organization (vision, mission, organizational chart, operations, geographical aspects), internal policies and regulations, employee benefits, systems, communication protocols, and company customs [103]. Likewise, Jarvis et al. emphasize among the key principles of induction the importance of clarity regarding job responsibilities, team dynamics, and organizational structure [104].

*“They gave me the opportunity to start, they gave me an orientation… the orientation period was only 2 or 3 shifts”.*

(Interview, Female nurse 1, ICUa, H1)

Studies in this field, such as that of McKenzie et al., explored the experiences of nurses newly assigned to a neonatal ICU, which were associated with psychosocial distress. This distress was exacerbated by a lack of structured support, horizontal violence, and inadequate or absent feedback from preceptors, all of which were the result of a negative work culture and inappropriate behavior from educators [105].

### 3.9. Career Development

Professional development includes opportunities for training, advancement, rewards, and recognition based on performance, as well as feedback and job evaluation systems [30,87]. Cox [21] identifies two potential sources of stress: job insecurity and obsolescence (fear of dismissal or early retirement), and status incongruence (under- or overpromotion and frustration at having reached a career ceiling).

*“You take diploma courses—I even have a Master’s degree—and they didn’t even acknowledge it, like officially recognizing my Master’s within the institution. So, there’s no incentive for professional growth, no monetary incentives either, no salary increases…”.*

(Interview, Female Nurse 3, ICUa, H1)

The data reflect a constant need for nurses to pursue training in order to meet the demands of their work. This need could be met through courses offered by institutions or through personal initiatives by the nurses themselves. Furthermore, nurses with specialized or Master’s degrees reported that their qualifications were not recognized by their institutions, perceiving a mismatch between the effort invested and the rewards received, which limited their professional development. This issue is particularly relevant in the studied context, as in Chile, despite a wide range of specialty training programs being available; only oncology nursing is formally regulated [106]. Additionally, some participants indicated that they had reached a “ceiling” in their careers, perceiving few opportunities for further growth.

*“I’m in a situation of… of uncertainty about the future, really… I’m studying a different field because I want to leave nursing—partly because I’m exhausted, and because I feel I’ve already hit a ceiling in this profession”.*

(Interview, Male Nurse 31, ICUa, H1)

*“I would never pursue a Master’s, because here it’s not like in other countries—here, you complete a diploma or Master’s and you earn the same…”.*

(Interview, Male Nurse 22, ICUa, H2)

These findings are consistent with the state of the art in the field. In the context of ICUs, there is a documented need for continuous scientific updating, in view of the technical and scientific advancements the specialty has undergone in recent years [76]. Such updating ensures that nurses’ competencies align with patient characteristics, thus facilitating synergistic and optimal outcomes [97]. Furthermore, recognizing nurses for their contributions has been highlighted as a key mechanism for sharing positive feedback and promoting engagement [97].

### 3.10. Institutional Strategic Planning

Institutional strategic planning refers to the series of stages through which senior leadership defines the direction and general guidelines that will govern the organization [107]. This planning process includes the institution’s philosophy, vision, mission, strategic objectives, strategies, policies, programs, and budget [108].

*“Macro-initiatives for the implementation of staff well-being strategies. Goal and periodicity: Equal to or greater than 80% compliance with the Quality of Life Plan”.*

(Document, Institutional Strategic Planning H1, Performance Dashboard–Staff Care Domain, 2025)

The data from this study revealed three main characteristics in this dimension. First, strategic planning documents did not always explicitly reflect a commitment to the reconciliation of work and personal roles, which meant that efforts toward reconciliation were often developed intuitively or implicitly by managerial staff. Second, national public policies had a direct influence—for example, in determining the nurse-to-patient ratio. Although this ratio was not consistently met, in the cases where it was, it tended to be at the upper limit of what is recommended by the Ministry of Health and was perceived as a barrier. Third, there was inconsistency between what was stated in strategic planning and what workers perceived in practice, such as with regard to flexible working hours in response to personal difficulties or exceptional circumstances, like the death of a family member.

*“Here, it’s six patients per nurse, but the pace is very fast—many discharges, patients who are very unstable… Staffing levels are low, right at the maximum threshold of what’s recommended by the Ministry of Health”.*

(Interview, Nurse Administrator 3, ICUa, H1)

These findings are consistent with the state of the art. A review that analyzed the potential impacts of work–life balance policies concluded that the development of such policies must be integrated with a culture of genuine support for reconciliation, as their mere availability and use do not appear to have consistent effects [109].

### 3.11. Public Policies

Adapting the definition proposed by Vargas Velásquez [110], public policies refer to the set of initiatives, decisions, and actions undertaken by a country, as well as the influence of international organizations, aimed at resolving or managing socially problematic issues. The conceptualization of this “Public Policies” dimension was informed by the theoretical framework for research on the psychosocial work environment and health developed by Rugulies [111], which describes the relationship between the psychosocial environment and phenomena at both the social and individual levels. Although Rugulies’ model is more detailed and specific, it follows the same logic of a macro-level dimension—referred to in the present theory as “Public Policies”—which influences the meso-level “Institutional Strategic Planning,” and this, in turn, affects the micro-level, comprising sources of interaction and the individual worker.

*“Supreme Decree No. 594, Basic Sanitary and Environmental Conditions in Workplaces, Article 3: The employer is obligated to maintain in workplaces the necessary sanitary and environmental conditions to protect the life and health of the workers performing duties therein, whether they are direct employees or third-party contractors providing services to the company”.*

(Document, Supreme Decree No. 594, Ministry of Health of Chile, 2000, p. 2)

Public policies associated with the phenomenon under study are present at various levels. At the international level, for example, they are embedded through the ratification of International Labour Organization (ILO) conventions such as Convention No. 156 on workers with family responsibilities [57,58]. At the national level, they are embedded in the regulatory framework—including the Constitution—which, for instance, recognizes the family as the fundamental unit of society [59]. They are also present at a more operational level, through national guidelines on how to assess and manage the phenomenon [61].

*“Recommended staffing levels for clinical nursing professionals (nurse-to-patient ratios): ICU patients (1:3) or (1:2) depending on the assessed care workload; specialized ICU (1:2); high-dependency ICU patients (1:1) or (2:1); Intermediate Treatment Unit (ITU) patients (1:6); highly specialized ITU (1:4)”.*

(Document, Operational Guidelines for Adult Critical Care Units, Ministry of Health of Chile, 2020, p. 54)

## 4. Strengths and Limitations

The limitations of this study are primarily framed within the socio-cultural context in which it was conducted, which may pose constraints on the transferability of the findings. Therefore, it is recommended that future studies provide evidence regarding the extent to which these findings can be transferred to other settings.

Regarding the strengths, this is, to the best of our knowledge, the first study to contribute to a comprehensive theorization of the phenomenon within the context of ICUs. Although it represents a theoretical proposal and is thus considered provisional, it offers a framework that facilitates the understanding of the work-related sources involved in the reconciliation of professional and personal roles, addressing a need previously identified by the scientific community [112]. The emerging proposal complements classical theories in the field, offering a comprehensive explanation of the sources of interaction of work roles and how these influence the underlying social process in the interaction between workers’ work and personal roles.

The present study generated a substantive theory, that is, a theory circumscribed to a particular context, derived from a systematic process in which data were elevated to a conceptual level; properties and their relationships were unveiled, all of which enabled the explanation of the reconciliation of work and personal roles, with clear applicability in contexts with similar characteristics.

Considering the recommendations in the field [30], the nine sources of interaction described in the theory can act as facilitators of reconciliation, such as the following: job content (providing psychological support in handling cases with high emotional demand and ensuring job variety); workload and pace (adequate workload organization and ensuring the necessary staffing levels); work schedule (flexible working hours, availability of a time bank when required, and compliance with departure times); control (active worker participation in decision-making, and control over workload and work pace); environment and equipment (a safe work environment, job design suited to workers’ needs, safe environmental conditions, and well-maintained equipment); organizational culture (promotion of a psychosocial safety climate, leadership awareness of psychosocial risks, and compliance with policies that facilitate work–life role reconciliation); interpersonal relationships (harmonious relationships among co-workers, supervisors, patients, and patients’ families); function within the organization (clarity of functions associated with the job position); and career development (skill development, effective feedback mechanisms, and opportunities for professional growth).

## 5. Conclusions

This article is part of a broader study that theorizes the work-related dimension involved in the process of reconciling professional and personal roles among nurses working in ICUs. Work roles are defined as the set of behaviors associated with the professional context of ICU nurses, which are shaped by nine sources of interaction: (1) Job content; (2) Workload and pace; (3) Work schedule; (4) Control; (5) Environment and equipment; (6) Organizational culture; (7) Interpersonal relationships; (8) Function within the organization; and (9) Career development. These sources of interaction are influenced by institutional strategic planning which, in turn, is shaped by national and international public policies. The theorization of work roles is essential for understanding the process of reconciling professional and personal roles among ICU nurses.

When the sources of interaction constitute a positive influence on workers, they contribute to the reconciliation of roles; when the sources of interaction exert a negative influence, the worker remains in a state of role conflict. In this regard, the proposed theory provides guidelines on management strategies for this significant occupational risk.

## Figures and Tables

**Table 1:** Demographic data of clinical nurses (*n* = 38) and nurse administrators (*n* = 8).

	Clinical Nurses (*n* = 38)	Nurse Administrators (*n* = 8)
Gender		
Male	10	0
Female	28	8
Age		
25–30	4	0
31–40	24	3
41–50	8	2
51–65	2	3
Unit *		
Intensive Care Units, adult (ICUa)	32	6
Intensive Care Units, pediatric/neonatal (ICUp)	6	2
Children/dependents under their care		
No	22	4
Yes	16	4
Work Experience (years)		
1–10	13	1
11–20	22	4
21–30	2	2
31–50	1	1
Experience in Current Position (years)		
1–10	31	1
11–20	6	7
21–30	1	0

* ICUa includes adult critical care units, both general and specialized (medical, surgical, burn, neurocritical, and coronary units); ICUp includes critical care units for pediatric and neonatal patients.

**Table 2:** Demographic data of nurses’ family members (*n* = 5).

Demographic Data	*n*
Gender	
Male	3
Female	2
Age	
18–30	1
31–40	3
41–50	1
Occupation	
Worker	4
Student	1

## Data Availability

For confidentiality purposes, the data are in the possession of the author (MVC).

## Supplementary Materials

The following supporting information can be downloaded at: https://www.mdpi.com/article/10.3390/healthcare13172134/s1, File S1: COREQ checklist; File S2: Data collection form; File S3: Coding process. Reference [46] is cited in Supplementary Materials.

## Abbreviations

The following abbreviations are used in this manuscript:

ICU	Intensive Care Unit

## References

[1] Geurts S.A.E., Taris T.W., Kompier M.A.J., Dikkers J.S.E., van Hooff M.L.M., Kinnunen U.M. (2005). Work-home interaction from a work psychological perspective: Development and validation of a new questionnaire, the SWING. Work Stress.

[2] Pujol-Cols L., Lazzaro-Salazar M. (2021). Ten years of research on psychosocial risks, health, and performance in Latin America: A comprehensive systematic review and research agenda. Rev. Psicol. Trab. Organ..

[3] Instituto Nacional de Seguridad y Salud en el Trabajo (2023). Conflicto trabajo-familia o doble presencia como riesgo psicosocial: Marco conceptual y consecuencias.

[4] Ratnaningsih I.Z., Idris M.A. (2024). Spillover–Crossover Effect of Work–Family Interface: A Systematic Review. Fam. J..

[5] Yang Q., Yang L., Yang C., Wu X., Xu Z., Wang X. (2025). How is work–family conflict linked to nurse-assessed patient safety among intensive care unit nurses? A serial multiple mediation analysis. Aust. Crit. Care.

[6] Yildiz B., Yildiz H., Ayaz Arda O. (2021). Relationship between work–family conflict and turnover intention in nurses: A meta-analytic review. J. Adv. Nurs..

[7] Toscano F., Tommasi F., Giusino D. (2022). Burnout in Intensive Care Nurses during the COVID-19 Pandemic: A Scoping Review on Its Prevalence and Risk and Protective Factors. Int. J. Environ. Res. Public Health.

[8] International Labour Organization (2020). Managing Work-Related Psychosocial Risks During the COVID-19 Pandemic.

[9] SUSESO (2024). Riesgo psicosocial laboral en Chile: Resultados de la aplicación del cuestionario de evaluación del ambiente laboral y salud mental 2023 CEAL-SM/SUSESO.

[10] Ekici D., Gurhan N., Mert T., Hizli I. (2020). Effects of work-to-family conflicts on services and individuals in healthcare professionals. Int. J. Caring Sci..

[11] Antolí-Jover A.M., Gázquez-López M., Brieba-del Río P., Pérez-Morente M.Á., Martín-Salvador A., Álvarez-Serrano M.A. (2024). Impact of work–family balance on nurses’ perceived quality of life during the COVID-19 pandemic: A scoping review. Nurs. Rep..

[12] Rendón-Díaz C., Vergas-Bertancourt M. (2019). El precio de la vocación en el personal de enfermería y su familia. Rev. Cubana Enferm..

[13] Ortega J. (2019). “Una cuestión de entrega”: Desigualdades de género y factores de riesgo psicosocial en el trabajo de enfermería. Soc. Cult..

[14] WHO (2020). State of the World’s Nursing 2020: Investing in Education, Jobs and Leadership.

[15] Rony M.K.K., Md. Numan S., Alamgir H.M. (2023). The association between work-life imbalance, employees’ unhappiness, work’s impact on family, and family impacts on work among nurses: A cross-sectional study. Inform. Med. Unlocked.

[16] Zhang A., Tao H., Ellenbecker C.H., Liu X. (2013). Job satisfaction in mainland China: Comparing critical care nurses and general ward nurses. J. Adv. Nurs..

[17] França D., Scaramuzzo P., Sacadura-Leite E. (2020). Psychosocial hazards evaluation in ICU workers. Occupational and Environmental Safety and Health II.

[18] Cooper C.L., Marshall J. (1976). Occupational sources of stress: A review of the literature relating to coronary heart disease and mental ill health. J. Occup. Psychol..

[19] Kasl S., Schroeder H. (1991). Assessing health risks in the work setting. New Directions in Health Psychology Assessment.

[20] Kasl S.V. (1998). Measuring job stressors and studying the health impact of the work environment: An epidemiologic commentary. J. Occup. Health Psychol..

[21] Cox T. (1993). Stress Research and Stress Management: Putting Theory to Work.

[22] Cox T., Cox S. (1993). Occupational health: Control and monitoring of psychosocial and organisational hazards at work. J. R. Soc. Health.

[23] Cox T., Griffiths A., Rial-González E. (2000). Research on Work-Related Stress.

[24] Cox T., Griffiths A., Randall R., Schabracq M., Winnubst J., Cooper C. (2003). A risk management approach to the prevention of work stress. The Handbook of Work and Health Psychology.

[25] Leka S., Cox T., Zwetsloot G., Leka S., Cox T. (2008). The European Framework for Psychosocial Risk Management (PRIMA-EF). The European Framework for Psychosocial Risk Management: PRIMA-EF.

[26] Leka S., Jain A. (2010). Health Impact of Psychosocial Hazards at Work: An Overview.

[27] van Stolk C., Staetsky L., Hassan E., Woo Kim C., Milczarek M., Irastorza X. (2012). Management of Psychosocial Risks at Work: An Analysis of the Findings of the European Survey of Enterprises on New and Emerging Risks (ESENER).

[28] Cox T. (2016). Managing psychosocial risks to worker health in the United Kingdom. Int. Labor Br..

[29] International Labour Organization (2016). Workplace Stress: A Collective Challenge.

[30] Leka S., Jain A. (2024). Conceptualising Work-Related Psychosocial Risks. Current State of the Art and Implications for Research, Policy and Practice.

[31] Neffa J.C. (2019). ¿Qué son los riesgos psicosociales en el trabajo?: Reflexiones a partir de una investigación sobre el sufrimiento en el trabajo emocional y de cuidado.

[32] Karasek R.A. (1979). Job demands, job decision latitude, and mental strain: Implications for job redesign. Adm. Sci. Q..

[33] Johnson J. (1986). The Impact of Workplace Social Support, Job Demands and Work Control. Ph.D. Thesis.

[34] Johnson J.V., Hall E.M. (1988). Job strain, workplace social support, and cardiovascular disease: A cross-sectional study of a random sample of the Swedish working population. Am. J. Public Health.

[35] Siegrist J. (1996). Adverse health effects of high-effort/low-reward conditions. J. Occup. Health Psychol..

[36] Siegrist J. (2017). The effort–reward imbalance model. The Handbook of Stress and Health: A Guide to Research and Practice.

[37] Valencia-Contrera M., Avilés L., Febré N. (2025). Reconciliation of work and personal roles among critical care nurses: Constructivist grounded theory research. Healthcare.

[38] Mihanovich C.S. (1945). Social processes according to von Wiese. Sociol. Rev..

[39] Palacios Rodríguez O. (2021). La teoría fundamentada: Origen, supuestos y perspectivas. Intersticios Soc..

[40] Charmaz K. (2017). Constructivist grounded theory. J. Posit. Psychol..

[41] Charmaz K. (2017). Special invited paper: Continuities, contradictions, and critical inquiry in grounded theory. Int. J. Qual. Methods.

[42] Charmaz K., Belgrave L.L. (2019). Thinking about data with grounded theory. Qual. Inq..

[43] Glaser B., Strauss A. (1967). The Discovery of Grounded Theory.

[44] Blumer H. (1982). El interaccionismo simbólico: Perspectiva y método.

[45] Charmaz K. (2014). Constructing Grounded Theory.

[46] Tong A., Sainsbury P., Craig J. (2007). Consolidated criteria for reporting qualitative research (COREQ): A 32-item checklist for interviews and focus groups. Int. J. Qual. Health Care.

[47] Valencia-Contrera M., Avilés L., Febré N. (2025). Theorizing the work–family interaction process in nurses in intensive care units: A constructivist grounded theory research protocol. Int. J. Qual. Methods.

[48] Instituto Nacional de Estadísticas (2024). Resultados Nacionales CENSO 2024.

[49] Mella P.F.O., Narváez C.G. (2022). Análisis del sistema de salud chileno y su estructura en la participación social. Saúde em Debate.

[50] Charmaz K. (2006). Constructing Grounded Theory: A Practical Guide Through Qualitative Analysis.

[51] Sadín Esteban M. (2003). Investigación cualitativa en educación: Fundamentos y tradiciones.

[52] Polit D., Beck C. (2018). Essentials of Nursing Research: Appraising Evidence for Nursing Practice.

[53] Sandoval Casilimas C. (2002). Investigación Cualitativa.

[54] Morse J., Clark L. (2019). The nuances of grounded theory sampling and the pivotal role of theoretical sampling. Current Developments in Grounded Theory.

[55] Farías L. (2016). La observación como herramienta de conocimiento y de intervención. Técnicas y estrategias en la investigación cualitativa.

[56] Martínez Trujillo N. (2020). La investigación cualitativa: Una mirada desde las ciencias sociales y humanas.

[57] ILO (1981). C156—Convention Concerning Workers with Family Responsibilities (No. 156).

[58] ILO (1983). Ratification of the C156—Convention Concerning Workers with Family Responsibilities, 1981 (No. 156).

[59] Ministerio Secretaría General de la Presidencia (2005). Decreto 100. Fija el texto refundido, coordinado y sistematizado de la constitución política de la república de Chile.

[60] Ministerio del Trabajo y Previsión Social, Subsecretaría de Previsión Social (1968). Ley 16.744: Establece normas sobre accidentes del trabajo y enfermedades profesionales.

[61] Ministerio de Salud, Subsecretaría de Salud Pública (2022). Resolución 1448 EXENTA.

[62] Ministerio de Salud Pública (1968). Decreto con Fuerza de Ley 725.

[63] Ministerio de Salud, Subsecretaría de Salud Pública (2012). Ley 20584|Regula los derechos y deberes que tienen las personas en relación con acciones vinculadas a su atención en salud.

[64] Instituto de Salud Pública (2022). Guía para la gestión de riesgos psicosociales en el trabajo: Equilibrio trabajo-vida privada.

[65] Subsecretaría de Redes Asistenciales (2020). Guía de funcionamiento y organización de Unidades de Pacientes Críticos Adultos.

[66] Subsecretaría de Redes Asistenciales (2022). Guía de diseño para establecimientos hospitalarios. Unidad de Pacientes Críticos.

[67] Strauss A., Corbin J. (2002). Bases de la investigación cualitativa: Técnicas y procedimientos para desarrollar la teoría fundamentada.

[68] Belgrave L., Seide K., Bryant A., Charmaz K. (2019). Coding for grounded theory. Current Developments in Grounded Theory.

[69] Nathaniel A. (2023). The logic and language of classic grounded theory: Induction, abduction, and deduction. Grounded Theory Rev..

[70] Charmaz K., Thornberg R. (2021). The pursuit of quality in grounded theory. Qual. Res. Psychol..

[71] Locke K. (2003). Writing grounded theory. Grounded Theory in Management Research.

[72] Biddle B. (1979). Glossary of key terms and concepts. Role Theory.

[73] Albizu Gallastegi E., Landeta Rodríguez J. (2013). Dirección estratégica de los recursos humanos: Teoría y práctica.

[74] National Health Service (2023). Intensive Care.

[75] Esin M.N., Sezgin D. (2017). Intensive Care Unit Workforce: Occupational Health and Safety.

[76] Aragão N.S.C., Barbosa G.B., Santos C.L.C., Nascimento D.S.S., Vilas Bôas L.B.S., Martins Júnior D.F., Nascimento Sobrinho C.L. (2021). Burnout syndrome and associated factors in intensive care unit nurses. Rev. Bras. Enferm..

[77] Chamberlain D., Pollock W., Fulbrook P. (2018). ACCCN workforce standards for intensive care nursing: Systematic and evidence review, development, and appraisal. Aust. Crit. Care.

[78] Detroja S., Mahajan R., Sheth A. (2025). Comprehensive investigation of ergonomic challenges and predictors of work-related musculoskeletal disorders among intensive care unit nurses of Western India through convergent mixed methods study. BMC Musculoskelet. Disord..

[79] Bloomer M.J., Brooks L.A., Coventry A., Ranse K., Rowe J., Thomas S. (2025). “You need to be supported”: An integrative review of nurses’ experiences after death in neonatal and pediatric intensive care. Aust. Crit. Care.

[80] Bloomer M.J., Ranse K., Adams L., Brooks L., Coventry A. (2023). “Time and life is fragile”: An integrative review of nurses’ experiences after patient death in adult critical care. Aust. Crit. Care.

[81] Almenyan A.A., Albuduh A., Al-Abbas F. (2021). Effect of nursing workload in intensive care units. Cureus.

[82] Salazar Roldán M.A., Venegas Paute A.C., Alarcón Dalgo C.M.d.L. (2024). Workload and performance obstacles of nursing staff in intensive care. Rev. Interdiscip. Cienc. Salud.

[83] Henao-Castano Á.M., Melo-Roa J.D., Quintero-Osorio J.F., Cruz-Lopez L.N. (2023). Nurses’ workload in intensive care unit according to the Nursing Activities Score. Rev. Cuid..

[84] Yan Y., Zhao C., Bi X., Or C.K., Ye X. (2025). The mental workload of ICU nurses performing human–machine tasks and associated factors: A cross-sectional questionnaire survey. J. Adv. Nurs..

[85] De Oliveira Salgado P., De Fátima Januário C., Vieira Toledo L., Miranda Brinati L., Sérvio de Araújo T., Tavares Boscarol G. (2020). Carga de trabalho da enfermagem requerida por pacientes durante internação numa UTI: Estudo de coorte. Enferm. Glob..

[86] Ahmadi N., Sasangohar F., Yang J., Yu D., Danesh V., Klahn S., Masud F. (2022). Quantifying workload and stress in intensive care unit nurses: Preliminary evaluation using continuous eye-tracking. Hum. Factors.

[87] Leka S., Jain A., Lerouge L. (2017). Work-related psychosocial risks: Key definitions and an overview of the policy context in Europe. Psychosocial Risks in Labour and Social Security Law Aligning Perspectives on Health, Safety and Well-Being.

[88] Ose S.O., Tjønnås M.S., Kaspersen S.L., Færevik H. (2019). One-year trial of 12-hour shifts in a non-intensive care unit and an intensive care unit in a public hospital: A qualitative study of 24 nurses’ experiences. BMJ Open.

[89] Webster J., McLeod K., O’Sullivan J., Bird L. (2019). Eight-hour versus 12-h shifts in an ICU: Comparison of nursing responses and patient outcomes. Aust. Crit. Care.

[90] Pruna Guanoluisa D.L. (2022). Condiciones de trabajo del personal de enfermería en la Unidad de Cuidados Intensivos. Sapienza Int. J. Interdiscip. Stud..

[91] Gurses A.P., Carayon P. (2009). Exploring performance obstacles of intensive care nurses. Appl. Ergon..

[92] Lewandowska K., Weisbrot M., Cieloszyk A., Mędrzycka-Dąbrowska W., Krupa S., Ozga D. (2020). Impact of alarm fatigue on the work of nurses in an intensive care environment—A systematic review. Int. J. Environ. Res. Public Health.

[93] Nyarko B.A., Yin Z., Chai X., Yue L. (2024). Nurses’ alarm fatigue, influencing factors, and its relationship with burnout in the critical care units: A cross-sectional study. Aust. Crit. Care.

[94] Uçak A., Cebeci F., Tat Çatal A. (2025). Nurses’ alarm fatigue levels in adult intensive care units and their strategies to reduce fatigue: A convergent parallel design. J. Clin. Nurs..

[95] Aldhafeeri N.A. (2024). Organizational culture: A concept analysis. Nurs. Sci. Q..

[96] Ritter M. (2008). Cultura organizacional: Gestión y comunicación.

[97] Browne C., Chun Tie Y. (2024). Promoting Well-being: A Scoping Review of Strategies Implemented During the COVID-19 Pandemic to Enhance the Well-being of the Nursing Workforce. Int. J. Nurs. Stud. Adv..

[98] Waddell A., Oberlies A.S. (2024). A post-pandemic review of American Association of Critical Care Nurses’s domains of establishing and sustaining healthy work environments: Strategies for nurse leaders. Crit. Care Nurs. Clin. N. Am..

[99] Sasaki H., Yonemoto N., Mori R., Nishida T., Kusuda S., Nakayama T. (2017). Assessing archetypes of organizational culture based on the Competing Values Framework: The experimental use of the framework in Japanese neonatal intensive care units. Int. J. Qual. Health Care.

[100] Teutsch D., Frick E., Kubitza J. (2025). What motivates critical care nurses to stay in their job? Structural aspects for empowering intrinsic motivation in permissive professional contexts: A scoping review. Intensive Crit. Care Nurs..

[101] Li X., Tian Y., Yang J., Ning M., Chen Z., Yu Q., Liu Y., Huang C., Li Y. (2025). Network of job demands-resources and depressive symptoms in critical care nurses: A nationwide cross-sectional study. Crit. Care.

[102] Adams A.M.N., Chamberlain D., Giles T.M. (2019). The perceived and experienced role of the nurse unit manager in supporting the well-being of intensive care unit nurses: An integrative literature review. Aust. Crit. Care.

[103] Alles M. (2000). Incorporación de candidatos. Dirección estratégica de recursos humanos: Gestión por competencias.

[104] Jarvis R., Word-Allen A., Jeffery A. (2024). Staff Educator’s Guide to Clinical Orientation.

[105] McKenzie R., Miller S., Cope V., Brand G. (2021). Transition experiences of newly qualified registered graduate nurses employed in a Neonatal Intensive Care Unit. Intensive Crit. Care Nurs..

[106] Ministerio de Salud, Subsecretaría de Redes Asistenciales (2013). Decreto 8. Reglamento de certificación de las especialidades de los prestadores individuales de salud y de las entidades que las otorgan.

[107] Münch L. (2011). Planeación estratégica: El rumbo hacia el éxito.

[108] Palacios Rodríguez M.Á. (2020). Planeación estratégica, instrumento funcional al interior de las organizaciones. Rev. Nac. Adm..

[109] Weale V., Oakman J., Wells Y. (2020). Can organisational work–life policies improve work–life interaction? A scoping review. Aust. Psychol..

[110] Vargas Velásquez A. (1999). El estado y las políticas públicas.

[111] Rugulies R. (2019). What is a psychosocial work environment?. Scand. J. Work Environ. Health.

[112] Chauhan P., Rai S. (2024). Conceptualizing work-life integration: A review and research agenda. Asia Pac. Manag. Rev..

