# Supplementary material for: Understanding the Work-Related Roles in the Work–Personal Life Reconciliation of Nurses in Intensive Care Units: Constructivist Grounded Theory Research
Source: Healthcare (Basel). 2025 Aug 27;13(17):2134. doi: 10.3390/healthcare13172134 (PMC12427950; doi:10.3390/healthcare13172134)
Supplement: Supplementary file 1 [file healthcare-13-02134-s001.zip › File S2.pdf]

DATA COLLECTION FORM

Non-Participant Observation

Date: ____ / ____ /202__ Time: _________

Location: ____

**Observation Specification**	**Description**
Individual and collective actions.	
Detailed notes, including anecdotes and observations.	
Emphasis on significant processes occurring in the environment.	
Consideration of what participants define as interesting and/or problematic.	
Attention to participants' language use.	
Contextualization of actors and actions within scenes and settings.	

Work charts: schemes, diagrams, and maps

IN-DEPTH INTERVIEWS

Date: ____ / ____ /202__ **Participation Code: ______________________**

**Sociodemographic Aspects**	
Would you like to be referred to by a pseudonym?	
What gender do you identify with?	
What is your date of birth?	
Do you have children or family members under your care?	
Considering all the jobs you have had throughout your life; how long have you been working in total?	
How long have you been working in your current unit (job)?	

**GUIDING QUESTIONS**		
**Nurses**	**Family Members of Nurses**	**Nurse Administrators**
-How is your routine on a day when you have to go to work?<br>-What are your thoughts on the relationship between work and family life?<br>-Tell me about a situation where you believe your work has influenced your family life.<br>-Tell me about a situation where you believe your family life has influenced your work.	-How is the routine on a day when your relative has to go to work?<br>-What are your thoughts on the relationship between work and family life?<br>-Tell me about a situation where you believe your relative's work has influenced family life.<br>-Tell me about a situation where you believe family life has influenced your relative's work.	-What are your thoughts on the relationship between work and family life of nursing staff?<br>-Tell me about a situation where you believe the work of nurses has influenced their family life.<br>-Tell me about a situation where you believe the family life of nurses has influenced their work.
Notes<br>____<br>____<br>____<br>____<br>____<br>____<br>____<br>____<br>____<br>____		
