# Supplementary material for: Understanding the Work-Related Roles in the Work–Personal Life Reconciliation of Nurses in Intensive Care Units: Constructivist Grounded Theory Research
Source: Healthcare (Basel). 2025 Aug 27;13(17):2134. doi: 10.3390/healthcare13172134 (PMC12427950; doi:10.3390/healthcare13172134)
Supplement: Supplementary file 1 [file healthcare-13-02134-s001.zip › File S3.pdf]

CODING PROCESS FOR THE DEVELOPMENT OF "WORK ROLES"

<table>
<tr><th>Data</th><th>Initial codes (n=133)</th><th>Theoretical links</th><th>Category and focused codes (n=50)</th></tr>
<tr><td>"Recommended staffing levels for clinical nursing professionals (nurse-to-patient ratios): ICU patients (1:3) or (1:2) depending on the assessed care workload; specialized ICU (1:2); high-dependency ICU patients (1:1) or (2:1); Intermediate Treatment Unit (ITU) patients (1:6); highly specialized ITU (1:4)". (Document, Operational Guidelines for Adult Critical Care Units, Ministry of Health of Chile, 2020, p. 54)</td><td rowspan="4">Work roles</td><td rowspan="4">What work elements are involved in the interaction? What are their characteristics?

Do the five sources of stress from the Cooper & Marshall (1976) model explain the work elements involved in the interaction?

What role do institutional policies play? What role do ministerial policies play?</td><td rowspan="4">-Public policies

-Institutional strategic planning

-Sources of interaction of work roles: (1) Job content; (2) Workload and pace; (3) Work schedule; (4) Control; (5) Environment and equipment; (6) Organizational culture; (7) Interpersonal relationships; (8) Function within the organization; and (9) Career development</td></tr>
<tr><td>"Here, it's six patients per nurse, but the pace is very fast—many discharges, patients who are very unstable… Staffing levels are low, right at the maximum threshold of what's recom-mended by the Ministry of Health". (Interview, Nurse Administrator 3, ICUa, H1)</td></tr>
<tr><td>"The shifts are very long. And on top of that, we often take extra shifts, so the commute adds another three hours to the job. That makes it 15 hours in a 24-hour day… it's hard…". (In-terview, Female Nurse 8, ICUa, H1)</td></tr>
<tr><td>"We take care of everything—respiratory, nutrition… we cover it all. Even things like making sure the unit doesn't run out of soap…". (Interview, Female Nurse 16, ICUa, H1)</td></tr>
</table>
